# Proteins at the intersection of circadian rhythms and metabolic dysfunction-associated steatotic liver disease: an 18-protein panel as a novel predictive biomarker set

**DOI:** 10.3389/fendo.2026.1836392

**Published:** 2026-05-13

**Authors:** Yiyi Wang, Qilong Zhai, Xuemei Lian, Lei Zhao

**Affiliations:** 1 Centre for Lipid Research & Chongqing Key Laboratory of Metabolism on Lipid and Glucose, Key Laboratory of Molecular Biology for Infectious Diseases (Ministry of Education), Institute for Viral Hepatitis, Department of Infectious Diseases, the Second Affiliated Hospital, Chongqing Medical University, Chongqing, China; 2 Chongqing Medical University-University of Leister Joint Institute, Chongqing Medical University, Chongqing, China; 3 School of Public Health, Chongqing Medical University, Chongqing, China

**Keywords:** circadian rhythm, machine learning, MASLD, prediction model, proteomics, UK Biobank

## Abstract

Disruption of circadian rhythmicity is highly prevalent in modern society and contributes to the epidemic of metabolic disorders. However, the relationship between circadian rhythm disorder and Metabolic Dysfunction-Associated Steatotic Liver (MASLD) remained poorly elucidated. Relative amplitude (RA), a metric quantifying the degree of disruption in rest–activity circadian rhythms, was calculated based on accelerometry data from UK Biobank. Basic characteristic analysis and multivariable logistic regression was used to analyze the association between RA and MASLD. Mediation analysis, functional enrichment analysis (Kyoto Encyclopedia of Genes and Genomes, KEGG) and Protein–Protein Interaction (PPI) network analysis and multiple machine learning algorithms, including Random Forest, XGBoost, logistic regression, and Support Vector Machine (SVM), were employed to identify potential protein biomarkers and construct a predictive model for RA-related MASLD risk assessment. Among 81,430 UK Biobank participants with valid accelerometry, RA was lower in individuals with MASLD versus those without (P < 0.001). Lower RA was associated to higher prevalence of MASLD (crude OR = 2.61; 95% CI [2.42, 2.81]; P < 0.001), and the association remained significant in a fully adjusted model (adjusted OR = 1.15; 95% CI [1.02,1.31]; P = 0.026), demonstrating RA as a factor independently associated with MASLD. Furthermore, 18 candidate proteins were identified as potential biomarkers for predicting RA-related MASLD. The 18-protein model demonstrated excellent predictive performance across multiple machine learning methods, with high Area Under the Curve (AUC) values in Receiver Operating Characteristic (ROC) analysis: Random Forest (AUC = 0.944), XGBoost (AUC = 0.946), Logistic Regression (AUC = 0.946), and SVM (AUC = 0.947). The model also exhibited superior discriminatory ability in predictive probability distribution, indicating strong predictive potential. Additionally, an online predictive tool based on this model has been contributed. Lower RA is independently associated with MASLD. We highlight 18 overlapping plasma proteins linked to both RA and MASLD as potential biomarkers for predicting RA-associated MASLD and as candidate therapeutic targets.

## Background

Metabolic Dysfunction-Associated Steatotic Liver Disease (MASLD), formerly known as Non-Alcoholic Fatty Liver Disease (NAFLD) has become one of the most prevalent chronic liver diseases worldwide. Its prevalence has increased from 25.26% (ranging from 21.59% to 29.33%) between 1990–2006 to 38.00% between 2016–2019, with an expected continued rise over the next decade (1). MASLD is associated with higher all-cause mortality and significantly elevates the risk of cirrhosis, hepatocellular carcinoma, and cardiovascular disease. Despite its widespread impact, pharmacological treatments for MASLD remain limited, and lifestyle modifications remain the primary evidence-based interventions. Among different modifications, adjusting the Circadian Rhythm (CR) appears to be a feasible approach.

CR is a biological process that fluctuates over a 24-hour period, regulated by a central pacemaker in the suprachiasmatic nucleus (SCN) and synchronized with peripheral clocks through complex neuronal and endocrine networks (2). CR integrates external cues, such as light, food, and exercise, with internal physiological processes, regulating diverse functions such as metabolism, body temperature, hormone secretion, immune function, and cell cycle progression (3, 4). This synchronization ensures efficient adaptation to changes in the external environment. In modern society, artificial lighting, constant temperatures, and sedentary lifestyles have disrupted circadian rhythms, leading to a range of pathologies, including diabetes, psychiatric disorders, immune impairment, and an increased risk of cancer (5). However, the relationship between the CR disruption and MASLD remains unclear in humans.

Relative Amplitude (RA), a key indicator of the CR, is an easily accessible, noninvasive, and behaviorally modifiable metric for assessing CR disruption. It reflects the intensity of the difference between high and low activity periods, effectively representing the strength of an individual’s circadian rhythm. RA has been widely adopted in previous experiments (6–12), and has been effectively utilized in psychiatric research (13–15). This provides a theoretical foundation for the future application of wearable devices in monitoring and evaluating disease states.

Recent studies have suggested that plasma proteins may serve as potential biomarkers for MASLD (16–18). Based on this, we hypothesize that RA, as a representative measure of circadian rhythm, may influence the plasma proteome and thereby contribute to the development of MASLD. To test this hypothesis and address the existing knowledge gap, we aim to investigate the association between the sleep-activity cycle and MASLD prevalence in the UK Biobank (UKB), which is one of the largest and most comprehensive biomedical research resources in the world. Furthermore, we aim to identify novel biomarkers and potential therapeutic targets for RA-related MASLD.

## Methods

### Study population and accelerometry data

UKB is a large-scale biomedical database that recruited more than 500,000 volunteers from across the United Kingdom between 2006 and 2010. Participants were recruited from all 22 assessment centers, donated biological samples, completed touchscreen questionnaires and underwent physiological monitoring.

For the present study, we included UKB participants with available data relevant to the assessment of MASLD. MASLD was defined as hepatic steatosis plus one of the following conditions: (1) type 2 diabetes mellitus, (2) obesity (body mass index ≥ 25 kg/m2), (3) metabolic abnormality including any of the two: insulin resistance, prediabetes (fasting glucose ≥ 100 mg/dl or hemoglobin A1c ≥ 5.7%), low high-density lipoprotein cholesterol (< 1.03 mmol/L for males: < 1.29 mmol/L for females), hypertriglyceridemia (≥ 1.7 mmol/L), hypertension (≥ 130/85 mmHg or use of antihypertensive medication), and increased waist circumference (≥ 102 cm for males; ≥ 88 cm for females).

Hepatic steatosis was assessed using the fatty liver index (FLI) as previously reported. Briefly, individuals with the fatty liver index (FLI) ≥ 60 were diagnosed hepatic steatosis. The FLI was calculated based on waist circumference (WC), body mass index (BMI), gamma-glutamyl transferase (GGT), and triglyceride (TG) levels, using the following formula: $FLI=\frac{e^{\left(0.953\times \ln(TG)+0.139\times BMI+0.718\times \ln(GGT)+0.053\times WC-15.745\right)}}{1+e^{\left(0.953\times \ln(TG)+0.139\times BMI+0.718\times \ln(GGT)+0.053\times WC-15.745\right)}}\times 100\;$ (21).

UKB participants were invited to wear a wrist-worn accelerometer continuously for 7 days while engaging in normal activities. A total of 103,720 participants successfully returned their devices. Preprocessing of the accelerometry data was conducted by the UK Biobank Accelerometry Expert Working Group. We extracted the hourly average acceleration (UKB Field ID: 90027–90050) to calculate the circadian rhythm metric RA.

To ensure data quality, we included only those with complete accelerometry records, and excluded individuals with insufficient wearing time (Field 90015) or uncalibrated data (Field 90016).

From a total of 502,147 participants in the UK Biobank, 160431 individuals had sufficient data available for the assessment of MASLD. Among these, 81,430 individuals with valid accelerometry data meeting wear-time and recalibration quality criteria were included as the primary cohort (Cohort 1).

**Figure 1:**
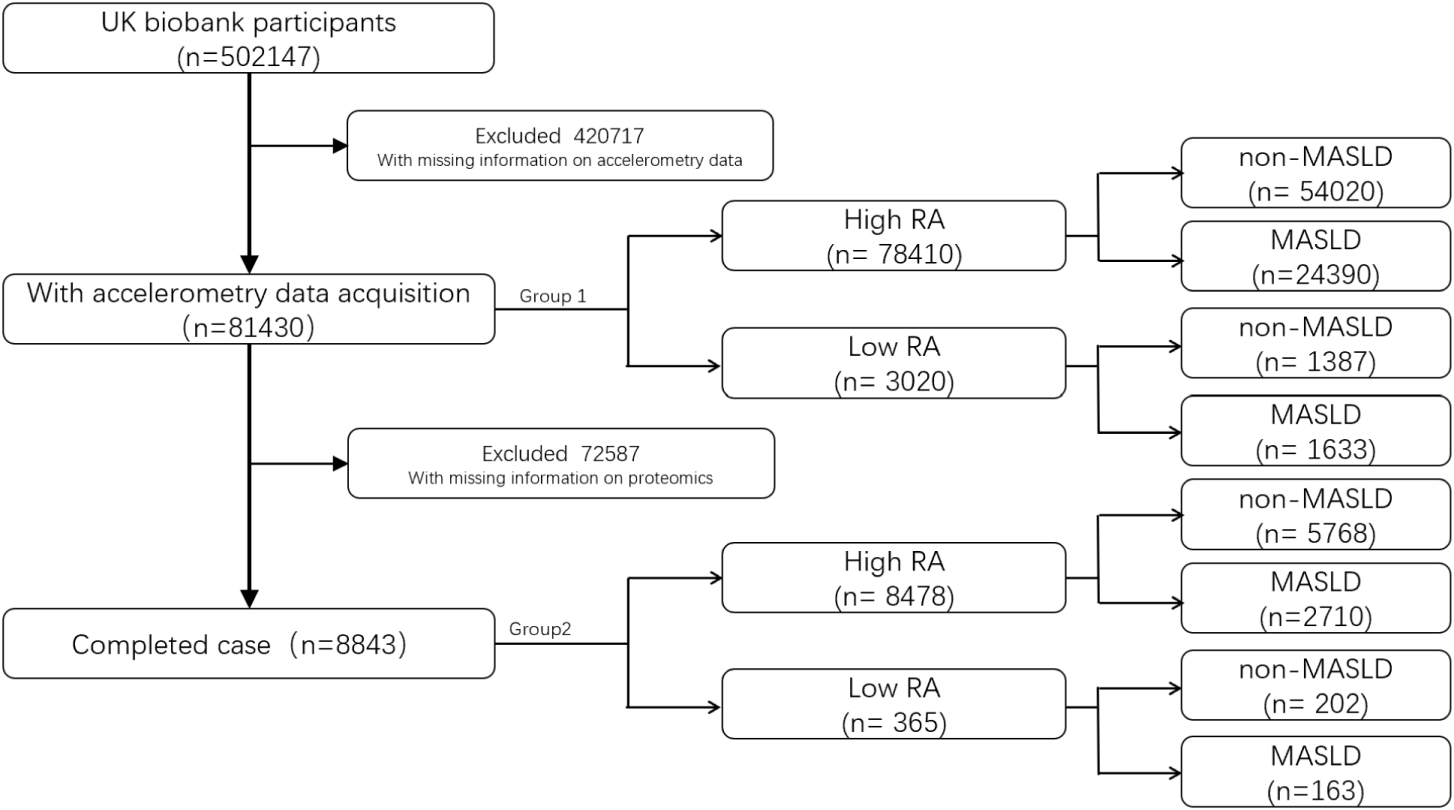
Study selection flowchart.

The study population was divided into two cohorts: Cohort 1 included all eligible participants after the above filtering; missing covariates were imputed, and cohort 2 was derived from Cohort 1 by excluding individuals without proteomics data, to facilitate subsequent protein-based analyses. A flow diagram of participant selection is provided in **Figure 1**.

### Relative amplitude

Relative Amplitude (RA) quantifies the difference in activity level between the most active 10-hour period (M10) and the least active 5-hour period (L5) within a 24-hour cycle, calculated using the formula:

Relative Amplitude = (M10 – L5)/(M10 + L5) (13).

M10 and L5 were derived from hourly average acceleration data (Fields 90027–90050). RA values range from 0 to 1, with values closer to 1 indicating greater disparity between active and resting periods, and values approaching 0 correspond to a reduction in the variability of activity levels. Lower RA reflects circadian rhythm disruption, which may result from reduced daytime activity, nocturnal sleep disturbances, or both. Based on prior literature, participants were classified into two groups: those with low relative amplitude (defined as > 2 standard deviations below the sample mean) and those with higher relative amplitude (all others) (13).

### Sleep behaviors, sleep score and sleep patterns

Sleep-related behaviors were collected via self-report including chronotype, sleep duration in hours per 24−h period, insomnia symptoms, snoring and daytime sleepiness. Sleep duration was categorized as short (<7 h/day), normal (7–8 h/day), and long (≥9 h/day) consistent with previous studies. A composite sleep score was constructed from the five sleep factors described above. Low−risk status for each factor was assigned according to the following criteria: early chronotype (i.e., choosing either of the first two options); sleeping 7–8 hours per day; reporting insomnia symptoms “never/rarely”; no snoring complaint; and daytime sleepiness occurring “never/rarely” or “sometimes”. For each factor, a value of 1 was given for low−risk and 0 for high−risk. Summing the five binary scores yielded a total ranging from 0 to 5, with higher totals reflecting more favorable sleep patterns. Based on this score, sleep patterns were categorized as healthy (score ≥4), intermediate (score 2–3), and poor (score ≤1) (19).

### Proteomics

Proteomic data were provided by the UK Biobank Pharma Proteomics Project (UKB-PPP). Plasma samples collected at baseline were analyzed using the Olink Explore Proximity Extension Assay platform, which measured 2,923 proteins. Detailed protocols regarding sample processing, plasma analysis using Olink technology, data handling, and quality control procedures have been comprehensively described in previous publications (20).

### Data analysis

For baseline characteristics, continuous variables were expressed as mean (standard deviation, SD) and compared using one-way ANOVA, while categorical variables were presented as frequency (percentage) and compared using the Chi-squared test. Logistic regression models were employed to evaluate the association between RA and the risk of MASLD. Models were adjusted for sociodemographic variables (including age, gender, education, ethnicity, Townsend Deprivation Index (TDI), drinking status, and smoking status) and metabolic-related variables (BMI and blood glucose).

### Proteomic profiling

First, differential protein expression analysis was performed between the circadian rhythm disruption group and the normal group, as well as between the MASLD group and the healthy group. Differential expression analysis was conducted using the “limma” R package, which fits linear models and applies empirical Bayes moderation to assess the association between circadian rhythm disruption and individual protein levels. Significantly differentially expressed proteins (DEPs) were identified based on adjusted p-values and visualized using volcano plots. The same procedure was repeated to evaluate the association between MASLD and protein expression.

Next, common DEPs across both comparisons were identified and visualized using Venn diagrams. Following the identification of proteins differentially expressed under varying circadian rhythms and MASLD conditions, mediation analysis was conducted using generalized linear models (logistic regression). Proteins with an ACME p-value < 0.05 were considered to have significant mediation effects.

Subsequently, a least absolute shrinkage and selection operator (Least Absolute Shrinkage and Selection Operator, LASSO)-penalized logistic regression model was applied to select proteins for inclusion in the final model and determine their weights. Proteins with a mediation proportion greater than 0.10 were included. The optimal regularization parameter λ was determined via 10-fold cross-validation based on minimum mean cross-validation error. Proteins with non-zero coefficients were retained, and a logistic regression model was constructed using these selected proteins and the optimal λ.

### KEGG pathway and PPI analysis

The ‘clusterProfiler’ R package was used to perform KEGG pathway enrichment analysis on the proteins selected by the LASSO model. Protein–protein interaction (PPI) analysis was conducted using the STRING database. Nodes within the top 10% of degree centrality were defined as “core” proteins. The most representative results were visualized using the ggraph R package.

### Machine learning predictive modeling

To quantitatively assess the relevance of selected proteins to MASLD onset, we developed predictive models using advanced machine learning techniques. The dataset was split into training and testing sets at a 7:3 ratio. A standard logistic regression model was trained using the generalized linear model function. Model performance was evaluated and visualized using ROC curves and AUC values.

The combination within 0.01 of the highest AUC value and with the fewest proteins was selected as optimal. And the top proteins up to the optimal number were identified as key proteins.

Using these key proteins, prediction models for MASLD were built and compared across four methods: ‘Random Forest’, ‘XGBoost’, ‘Logistic Regression’, and ‘SVM’. Stratified sampling by the outcome variable was performed using ‘createDataPartition’, with 70% of data for training and 30% for testing. Model performance was evaluated using ‘twoClassSummary**’** with AUC as the primary metric.

All analyses were performed using R (version 4.4.2) and RStudio, with the following R packages: “AnnotationDbi”, “boot”, “broom”, “caret”, “clusterProfiler”, “doParallel”, “dplyr”, “FastUKB”, “foreach”, “ggraph”, “ggplot2”, “ggrepel”, “ggvenn”, “glmnet”, “grid”, “igraph”, “iterators”, “limma”, “mediation”, “org.Hs.eg.db”, “parallel”, “patchwork”, “pROC”, “randomForest”, “shiny”, “STRINGdb”, “tableone”, “tidygraph”, “tidyverse”, “xgboost”. A two-tailed p-value < 0.05 was considered statistically significant.

## Results

### RA is reduced in individual with MASLD in UKB

The study population consisted of 81,430 participants. The mean age was 56.18 years (standard deviation: 7.81, range: 40-49), and 44.25% were male.

Basic characteristic analysis showed that the MASLD group had a significantly higher proportion of males and older individuals compared to the non-MASLD group.

The MASLD group exhibited higher levels of Townsend Deprivation Index (TDI), Body Mass Index (BMI), Systolic Blood Pressure (SBP), Diastolic Blood Pressure (DBP), Hemoglobin A1c (HbA1c), fasting glucose, Low-Density Lipoprotein Cholesterol (LDL-C), triglycerides (TG), and C-reactive Protein (CRP), but lower High-Density Lipoprotein Cholesterol (HDL-C) levels. A history of smoking or drinking with lower education levels was more common in MASLD group. Notably, RA was significantly lower in the MASLD group compared to the non-MASLD group. In addition, compared with individuals without MASLD, those with MASLD showed significantly higher proportions of insufficient sleep (< 7 h) and higher prevalence of poor sleep patterns, as defined by the composite sleep score (**Table 1**).

**Table 1:** Baseline characteristics of the overall population, stratified by MASLD and non-MASLD.

Characteristics	Non-MASLD	MASLD	P
Group number	55407	26023	
age (mean (SD))	55.76 (7.91)	57.07 (7.52)	<0.001
gender = Male (%)	18953 (34.2)	17082 (65.6)	<0.001
ethnicity = White (%)	53707 (97.2)	25222 (97.3)	0.748
TDI (mean (SD))	-1.82 (2.77)	-1.58 (2.89)	<0.001
education (%)			<0.001
College	25634 (46.3)	9470 (36.4)	
Other levels	25636 (46.3)	13545 (52.1)	
Unknown	4137 (7.5)	3008 (11.6)	
drinking status (%)			<0.001
Current	52348 (94.6)	24482 (94.2)	
Never	1587 (2.9)	708 (2.7)	
Previous	1425 (2.6)	811 (3.1)	
Smoking status (%)			<0.001
Current	3452 (6.2)	2185 (8.4)	
Never	33583 (60.8)	12650 (48.8)	
Previous	18240 (33.0)	11112 (42.8)	
BMI* (mean (SD))	2.46 (0.57)	3.52 (0.54)	<0.001
SBP	135.03 (17.49)	143.33 (16.87)	<0.001
DBP	80.93 (9.51)	86.50 (9.67)	<0.001
Fasting glucose	4.96 (0.82)	5.27 (1.38)	<0.001
HbA1C	34.58 (4.30)	37.09 (7.19)	<0.001
LDL-C	3.52 (0.82)	3.68 (0.90)	<0.001
HDL-C	1.59 (0.38)	1.26 (0.29)	<0.001
TG	1.32 (0.61)	2.39 (1.15)	<0.001
CRP	1.74 (3.43)	3.33 (4.55)	<0.001
RA (mean (SD))	0.87 (0.06)	0.84 (0.08)	<0.001
obesity	0.43 (0.50)	0.98 (0.15)	<0.001
T2DM	0.01 (0.09)	0.05 (0.22)	<0.001
Sleep duration status (%)			<0.001
<=5h	1965 (3.5)	1352 (5.2)	
6h	9485 (17.1)	5175 (19.9)	
7h	24190 (43.7)	10387 (39.9)	
8h	16556 (29.9)	7190 (27.6)	
>=9h	3191 (5.8)	1912 (7.3)	
score pattern (%)			<0.001
health	34020 (61.4)	11988 (46.1)	
moderate	20322 (36.7)	12882 (49.5)	
poor	1065 (1.9)	1153 (4.4)	

*, BMI levels: 1: <18.5; 2: >=18.5 and <25; 3:>= 25 and <30; 4: >=30 (35).

MASLD, Metabolic dysfunction-associated steatotic liver disease; P value, probability value; TDI, Townsend Deprivation Index; BMI, Body Mass Index; SBP, Systolic Blood Pressure; DBP, Diastolic Blood Pressure; HbA1c, Hemoglobin A1c; LDL-C, Low-Density Lipoprotein Cholesterol; HDL-C, High-Density Lipoprotein Cholesterol; TG, triglyceride; CRP, C-reactive Protein; RA, Relative Amplitude; T2DM, Type 2 Diabetes. Mellitus.

Next, we also stratified the population into two groups based on RA levels, for a baseline assessment. Individuals with low RA exhibited significantly higher proportions of insufficient sleep (<7 h) and greater prevalence of poor sleep patterns compared with those with high RA. The low RA group had a lower mean age compared to the healthy population (55.41 years vs. 56.21 years) and a higher proportion of males (59.8% vs. 43.7%). Among the defined risk factors for MASLD, the FLI value was significantly higher in the low RA group, along with a greater proportion of individuals with a history of T2DM (7% vs. 2%) and a likewise higher proportion of obese individuals (77% vs. 60%) (**Table 2**). We divided the participants into four groups based on RA and MASLD status. Baseline characteristics of the four groups are presented in **Table 3**. Beside the lower age and higher proportion of males, the low RA group also had a lower proportion of White individuals with lower education levels and more current smoker and less current drinker. Additionally, across the four groups, the low RA group showed higher levels of TDI, BMI, HbA1c, CRP, but lower SBP, DBP, total cholesterol, HDL-C and LDL-C (**Table 3**). However, when only comparing the high-RA and low-RA groups (**Table 2**), SBP and DBP showed no significant difference.

**Table 2:** Baseline characteristics of the overall population, stratified by low and high RA values.

Characteristics	High RA	Low RA	P
Group number	78410	3020	
age (mean (SD))	56.21 (7.79)	55.41 (8.33)	<0.001
gender = Male (%)	34230 (43.7)	1805 (59.8)	<0.001
ethnicity = White (%)	76134 (97.4)	2795 (93.1)	<0.001
TDI (mean (SD))	-1.78 (2.78)	-0.71 (3.33)	<0.001
education (%)			<0.001
College	33959 (43.3)	1145 (37.9)	
Other levels	37617 (48.0)	1564 (51.8)	
Unknown	6834 (8.7)	311 (10.3)	
drinking status (%)			<0.001
Current	74141 (94.6)	2689 (89.2)	
Never	2147 (2.7)	148 (4.9)	
Previous	2057 (2.6)	179 (5.9)	
Smoking status (%)			<0.001
Current	5226 (6.7)	411 (13.6)	
Never	44760 (57.2)	1473 (48.9)	
Previous	28225 (36.1)	1127 (37.4)	
BMI* (mean (SD))	2.78 (0.75)	3.15 (0.78)	<0.001
SBP	137.59 (17.75)	137.17 (17.03)	0.771
DBP	82.65 (9.90)	82.37 (9.93)	0.727
Fasting glucose	5.05 (1.01)	5.31 (1.69)	<0.001
HbA1C	35.30 (5.34)	37.42 (8.82)	<0.001
LDL-C	3.58 (0.85)	3.44 (0.88)	<0.001
HDL-C	1.49 (0.38)	1.33 (0.36)	<0.001
TG	1.65 (0.95)	1.91 (1.10)	<0.001
CRP	2.21 (3.84)	3.28 (5.07)	<0.001
RA (mean (SD))	0.87 (0.05)	0.61 (0.12)	<0.001
obesity	0.60 (0.49)	0.77 (0.42)	<0.001
T2DM	0.02 (0.14)	0.07 (0.26)	<0.001
Sleep duration status (%)			<0.001
<=5h	3019 (3.9)	298 (9.9)	
6h	13893 (17.7)	767 (25.4)	
7h	33519 (42.8)	1058 (35.1)	
8h	23078 (29.4)	668 (22.1)	
>=9h	4876 (6.2)	227 (7.5)	
score pattern (%)			<0.001
health	44783 (57.1)	1225 (40.6)	
moderate	31617 (40.3)	1587 (52.5)	
poor	2010 (2.6)	208 (6.9)	

*, BMI levels: 1: <18.5; 2: >=18.5 and <25; 3: >= 25 and <30; 4: >=30 (35).

MASLD, Metabolic dysfunction-associated steatotic liver disease; P value, probability value; TDI, Townsend Deprivation Index; BMI, Body Mass Index; SBP, Systolic Blood Pressure; DBP, Diastolic Blood Pressure; HbA1c, Hemoglobin A1c; LDL-C, Low-Density Lipoprotein Cholesterol; HDL-C, High-Density Lipoprotein Cholesterol; TG, triglyceride; CRP, C-reactive Protein; RA, Relative Amplitude; T2DM, Type 2 Diabetes Mellitus.

**Table 3:** Baseline characteristics of the overall population, stratified by MASLD and non-MASLD with low and high RA values.

Characteristics	High RA		Low RA		p
	Non-MASLD	MASLD	Non-MASLD	MASLD	
Group number	54020	24390	1387	1633	
age (mean (SD))	55.81 (7.89)	57.09 (7.48)	53.89 (8.37)	56.70 (8.07)	<0.001
gender = Male (%)	18284 (33.8)	15946 (65.4)	669 (48.2)	1136 (69.6)	<0.001
ethnicity = White (%)	52445 (97.4)	23689 (97.5)	1262 (91.4)	1533 (94.5)	<0.001
TDI (mean (SD))	-1.84 (2.75)	-1.66 (2.84)	-0.98 (3.23)	-0.48 (3.39)	<0.001
education (%)					<0.001
College	25035 (46.3)	8924 (36.6)	599 (43.2)	546 (33.4)	
Other levels	24943 (46.2)	12674 (52.0)	693 (50.0)	871 (53.3)	
Unknown	4042 (7.5)	2792 (11.4)	95 (6.8)	216 (13.2)	
drinking status (%)					<0.001
Current	51109 (94.7)	23032 (94.5)	1239 (89.5)	1450 (88.9)	
Never	1514 (2.8)	633 (2.6)	73 (5.3)	75 (4.6)	
Previous	1352 (2.5)	705 (2.9)	73 (5.3)	106 (6.5)	
Smoking status (%)					<0.001
Current	3251 (6.0)	1975 (8.1)	201 (14.5)	210 (12.9)	
Never	32826 (60.9)	11934 (49.1)	757 (54.7)	716 (44.0)	
Previous	17813 (33.1)	10412 (42.8)	427 (30.8)	700 (43.1)	
BMI* (mean (SD))	2.46 (0.57)	3.51 (0.54)	2.56 (0.61)	3.66 (0.50)	<0.001
SBP	135.07 (17.57)	143.49 (16.75)	133.62 (14.79)	140.90 (18.47)	<0.001
DBP	80.94 (9.52)	86.66 (9.60)	80.67 (9.17)	84.16 (10.45)	<0.001
Fasting glucose	4.96 (0.82)	5.25 (1.32)	4.95 (0.86)	5.60 (2.10)	<0.001
HbA1C	34.57 (4.27)	36.93 (6.89)	34.92 (5.24)	39.51 (10.50)	<0.001
LDL-C	3.52 (0.82)	3.70 (0.90)	3.40 (0.83)	3.48 (0.91)	<0.001
HDL-C	1.60 (0.38)	1.27 (0.29)	1.49 (0.38)	1.21 (0.29)	<0.001
TG	1.32 (0.61)	2.39 (1.14)	1.36 (0.65)	2.39 (1.19)	<0.001
CRP	1.73 (3.41)	3.27 (4.46)	2.13 (4.00)	4.27 (5.65)	<0.001
RA (mean (SD))	0.87 (0.04)	0.85 (0.05)	0.60 (0.13)	0.62 (0.11)	<0.001
Obesity	0.43 (0.50)	0.98 (0.15)	0.52 (0.50)	0.99 (0.12)	<0.001
T2DM	0.01 (0.09)	0.05 (0.21)	0.02 (0.15)	0.12 (0.32)	<0.001

*, BMI levels: 1: <18.5; 2: >=18.5 and <25; 3:>= 25 and <30; 4: >=30 (35).

MASLD, Metabolic dysfunction-associated steatotic liver disease; P value, probability value; TDI, Townsend Deprivation Index; BMI, Body Mass Index; SBP, Systolic Blood Pressure; DBP, Diastolic Blood Pressure; HbA1c, Hemoglobin A1c; LDL-C, Low-Density Lipoprotein Cholesterol; HDL-C, High-Density Lipoprotein Cholesterol; TG, triglyceride; CRP, C-reactive Protein; RA, Relative Amplitude; T2DM, Type 2 Diabetes Mellitus.

### The reduced RA is a factor independently associated with MASLD

We employed logistic regression to assess the association between RA and MASLD. In the crude model, lower RA was associated with higher odds of MASLD (odds ratio [OR] = 2.61; 95% CI [2.42, 2.81]; P < 0.001). This association remained significant after multivariable adjustment (OR = 1.15; 95% CI [1.02, 1.31]; P = 0.026), indicating that RA is a factor independently associated with MASLD (**Table 4**).

**Table 4:** Association between relative amplitude and MASLD in multivariable logistic regression models.

	Variables	OR	95%CI	P value
Crude model	RA	2.61	(2.42, 2.81)	<0.001
Adjusted model1	RA, age, gender	2.35	(2.17, 2.55)	<0.001
Adjusted model2	RA, age, gender, education, ethnicity, TDI	2.22	(2.05, 2.40)	<0.001
Adjusted model3	RA, age, gender, education, ethnicity, TDI, Glucose, drinking status, smoking status	1.15	(1.01, 1.31)	=0.026

MASLD, Metabolic dysfunction-associated steatotic liver disease; OR, odds ratio; CI, confidence interval; P value, probability value; RA, Relative Amplitude; TDI, Townsend Deprivation Index.**].**

During adjustment analyses, BMI was the strongest covariate between circadian rhythm and MASLD (OR = 2.17), with each 1-unit increase nearly doubling MASLD risk. Males had approximately 10 times higher risk than females (OR = 10.21). Advanced age, elevated blood glucose, and lower education level were also significant risk factors.

### Screening candidate proteins associated with RA-related MASLD

Plasma proteomics is commonly employed in medical research for biomarker discovery. Hence, from Cohort 1, we selected participants with available proteomics data to form Cohort 2 for subsequent analysis. Cohort 2 included 8,843 participants with a mean age of 56.07 years; 43.96% were male. The MASLD subgroup within this cohort demonstrated a higher proportion of males and older participants compared to the non-MASLD subgroup. Other baseline characteristics were largely consistent with those reported in Cohort 1.

**Figure 2:**
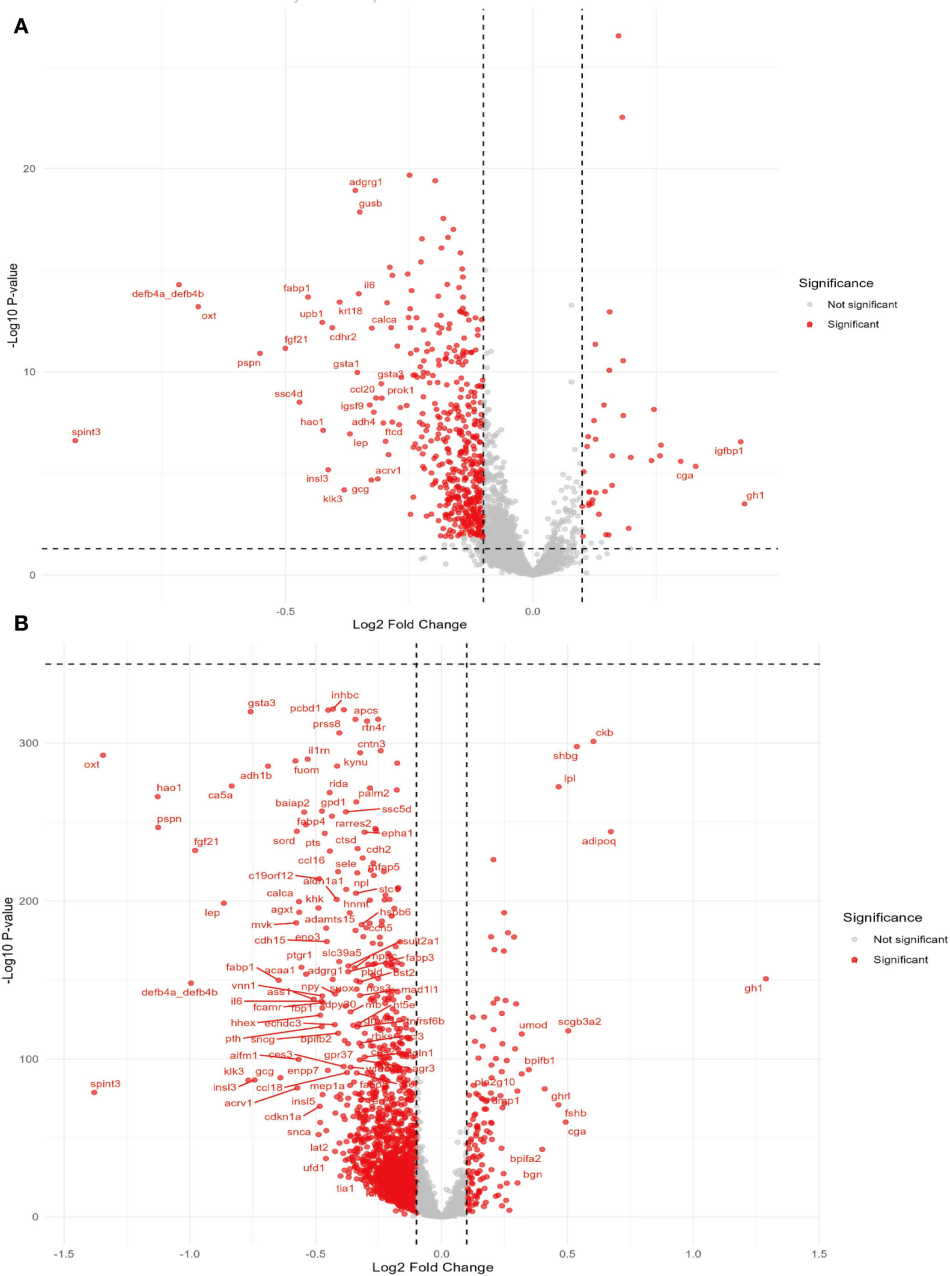
Volcano plot: displaying differential expressed proteins in circadian rhythm disruption and MASLD. **(A)** Differential proteins in circadian rhythm disruption. **(B)** Differential proteins in MASLD.

Through variance analysis with RA and MASLD, we identified 494 proteins associated with RA and 1427 proteins associated with MASLD, respectively. The intersection of these sets revealed 453 DEPs significantly associated with both conditions. These DEPs visualized using volcano plots and Venn diagram (**Figure 2**). We subsequently performed mediation analysis and constructed a preliminary predictive model via LASSO regression, which selected features with non-zero coefficients to narrow down the number of protein variables for downstream modeling. This process identified 145 proteins exhibiting significant mediating effects.

### Construction of a protein interaction network

**Figure 3:**
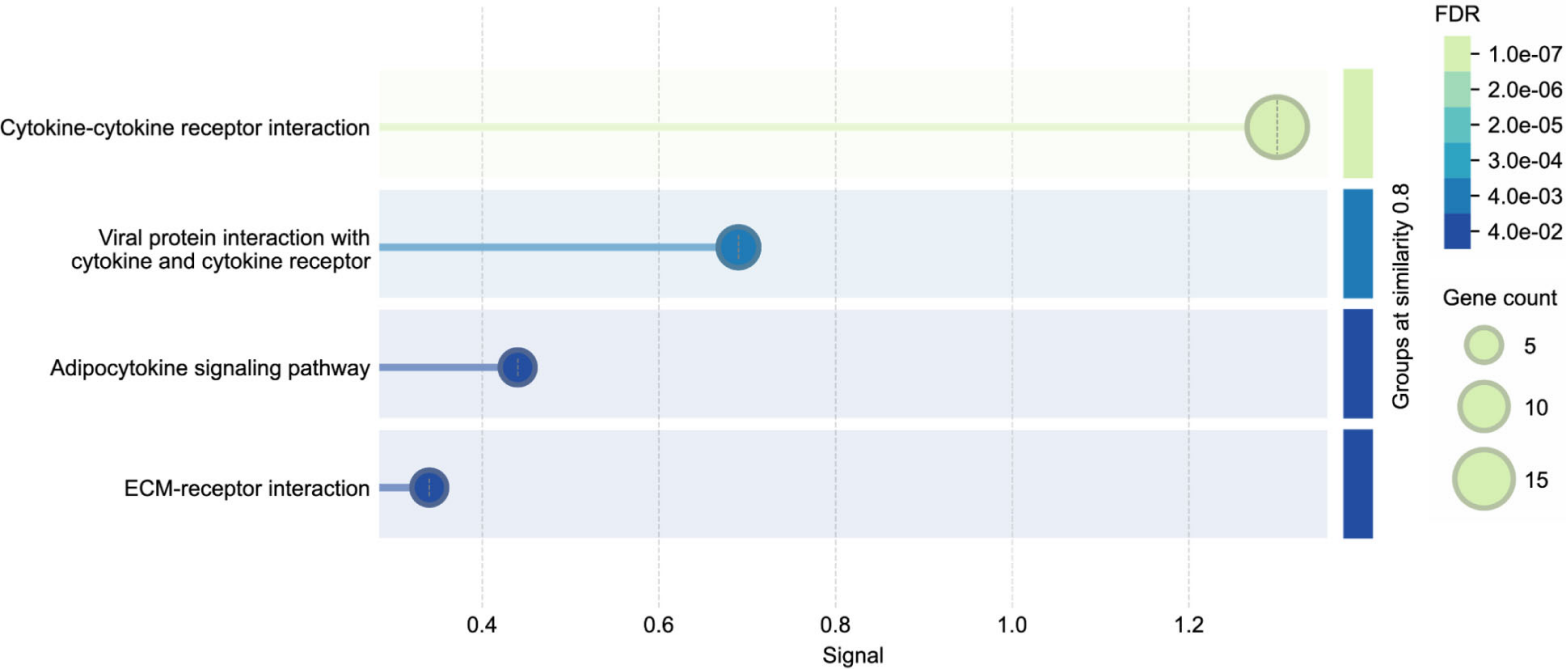
Enrichment pathway of KEGG.

**Figure 4:**
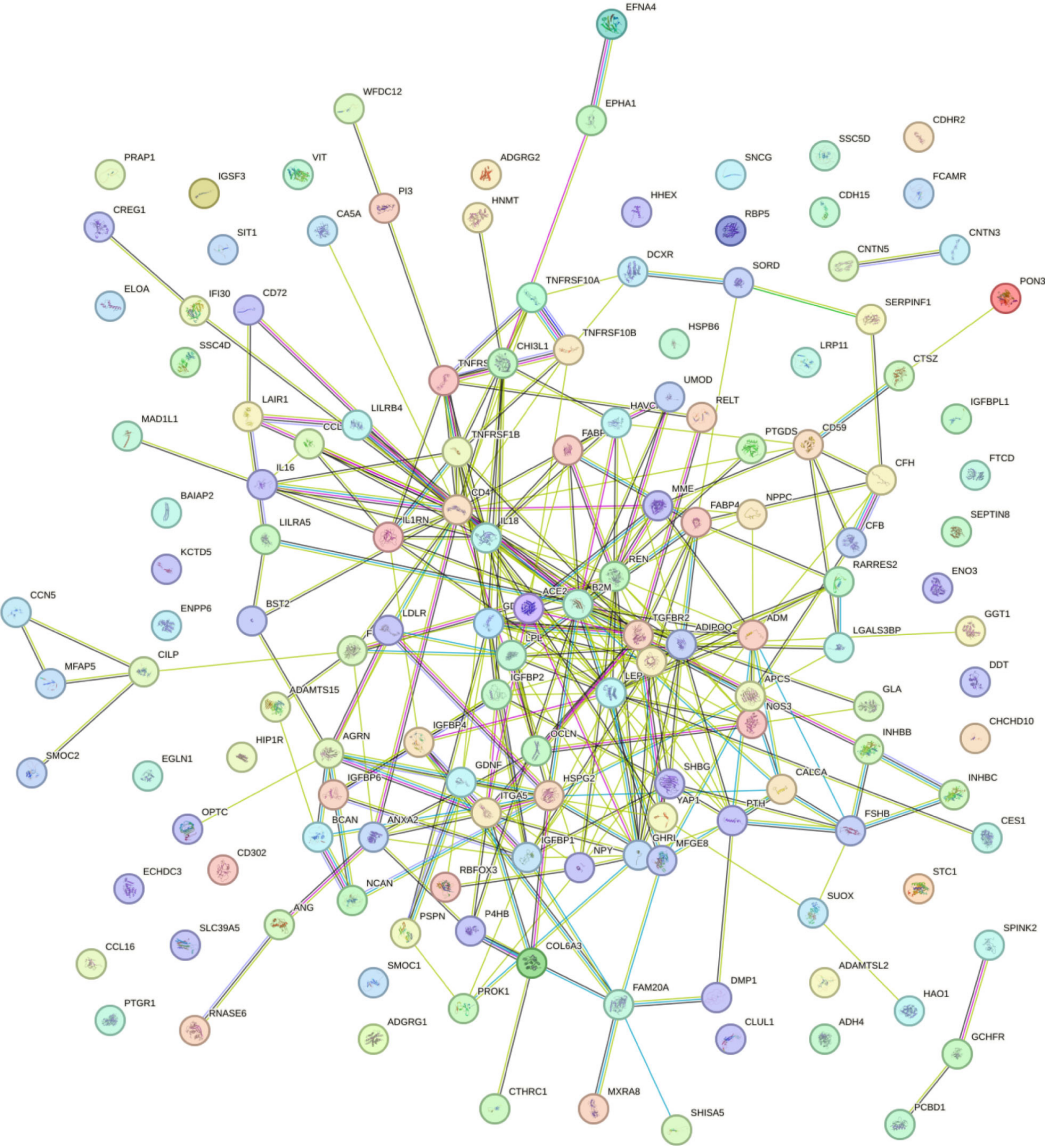
PPI analysis result.

KEGG pathway analysis was conducted on the key proteins identified by the LASSO model to elucidate the biological functions of the DEPs. The main enriched pathways are cytokine-cytokine receptor interaction and lipid metabolism pathways (**Figure 3**), the latter of which is a well-established mechanism in MASLD pathogenesis. Additionally, protein-protein interaction (PPI) analysis was performed, hub proteins were explored, and potential molecular targets were obtained. A representative subset of interactions was visualized in **Figure 4**.

### Validation and application of biomarkers for RA-related MASLD

**Figure 5:**
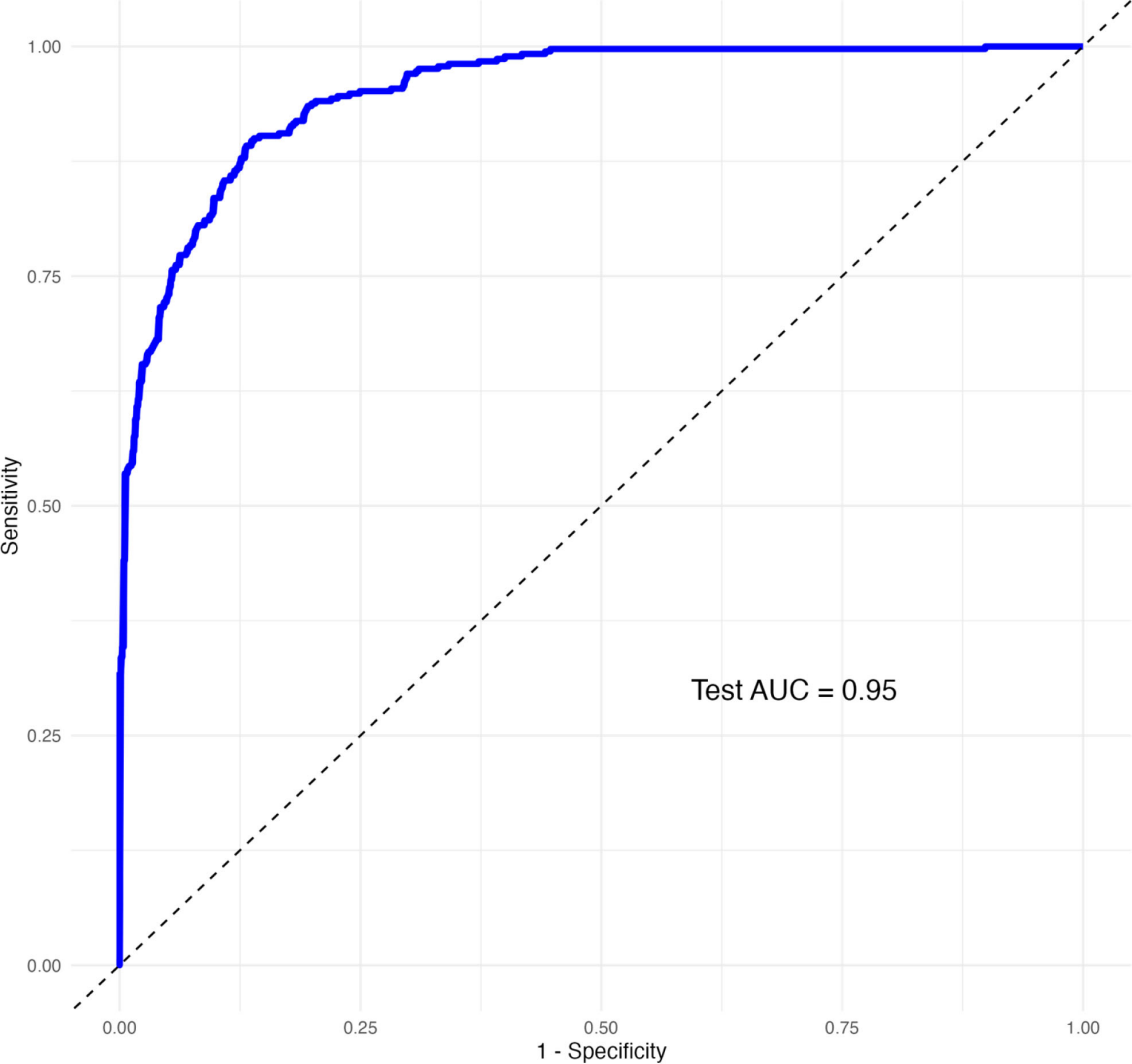
The preliminary ROC carve generated by 145 proteins.

**Figure 6:**
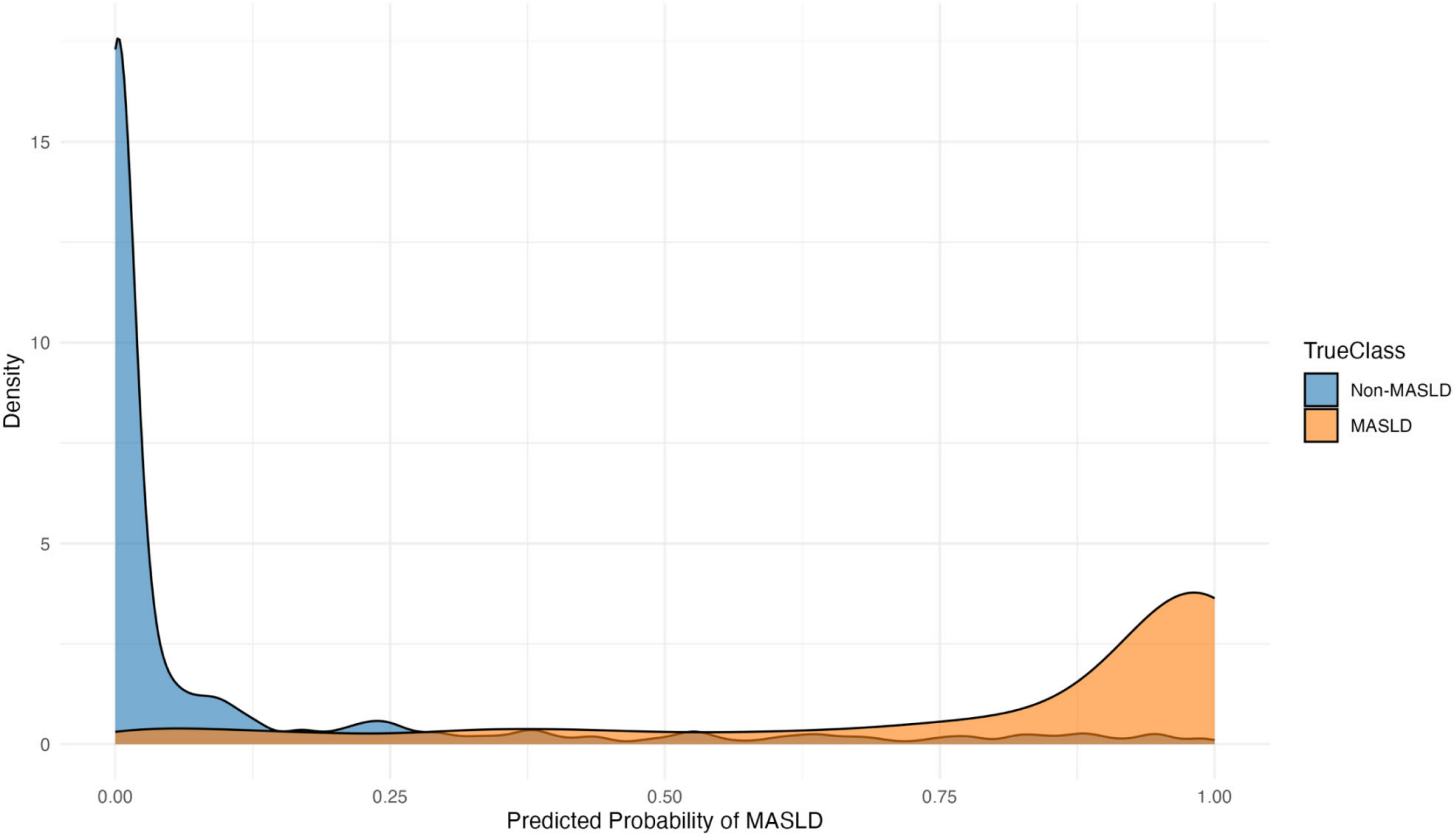
The preliminary predicted probability distribution generated by 145 proteins.

**Figure 7:**
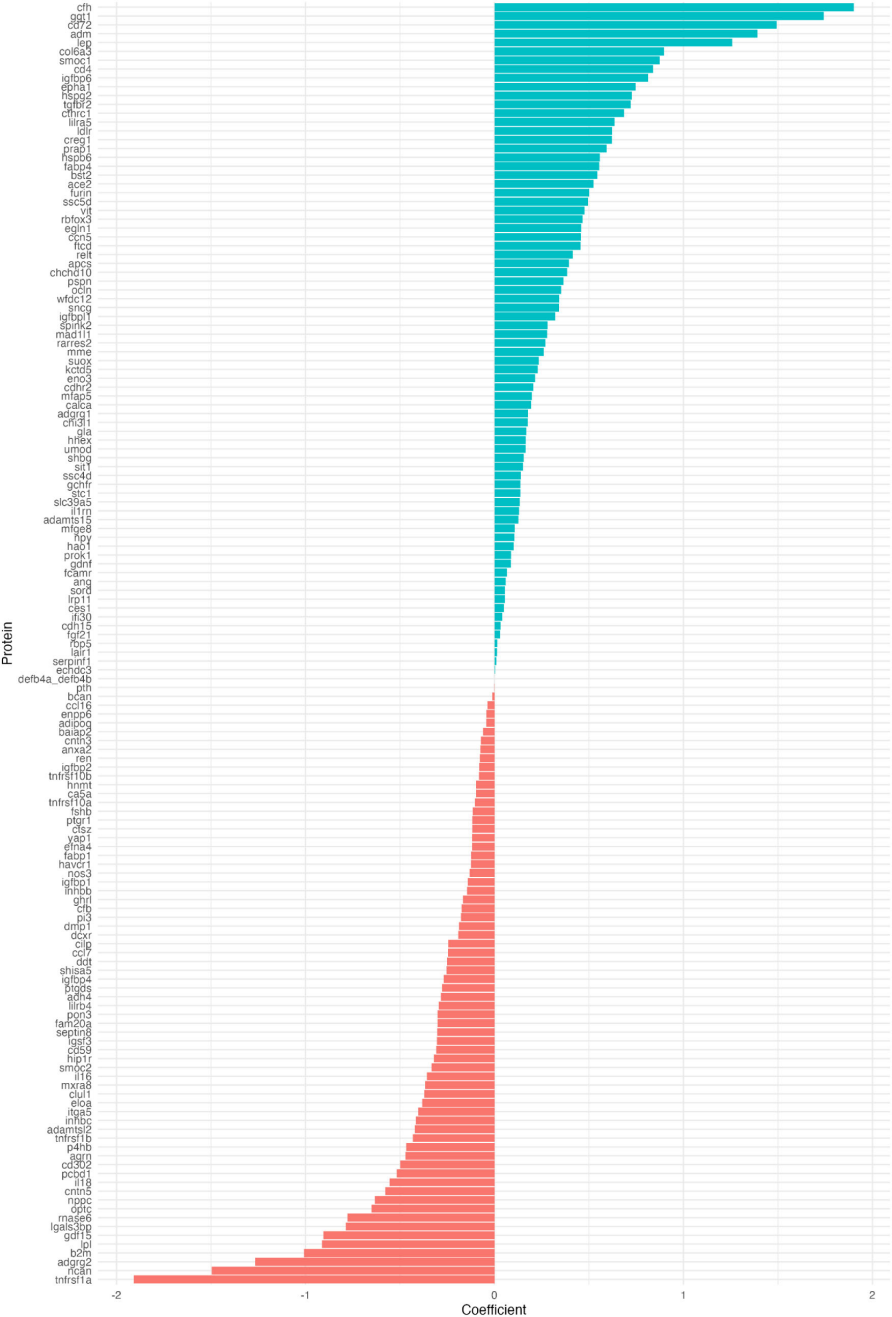
Protein coefficients in prediction model.

To enable internal validation, the cohort was split into training (70%) and testing (30%) sets. A logistic regression model was trained using a standard generalized linear model function and incorporating all 145 proteins. Predicted probability distribution and ROC curve were used to verified the predictive performance of the 145-protein model. The model achieved an area under the curve (AUC) of 0.95 (**Figure 5**). The predicted probability distributions showed a clear separation in central tendencies between MASLD and non-MASLD groups, indicating a moderate discriminatory ability with substantial overlap was also observed (**Figure 6**). Furthermore, all 145 selected proteins were ranked in descending order based on their λ values (**Figure 7**). Proteins with λ values closer to zero exhibited smaller effect sizes and contributed less to the model’s predictive performance.

**Figure 8:**
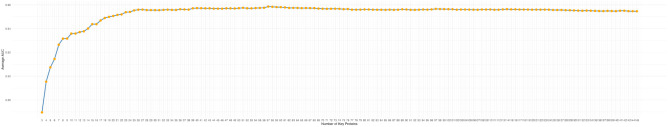
Model performance vs number of proteins.

To optimize the trade-off between performance and practicality, we aimed to minimize the number of proteins while limiting the decline in AUC to less than 0.01 (**Figure 8**). This resulted in a refined model based on 18 key proteins: neurocan (NCAN) protein, gamma-glutamyltransferase 1 (GGT1) protein, adrenomedullin (ADM) protein, Leptin (LEP) protein, low density lipoprotein receptor (LDLR) protein, opticin (OPTC) protein, lipoprotein lipase (LPL) protein, adhesion G protein-coupled receptor G2 (ADGRG2) protein, growth differentiation factor 15 (GDF15) protein, heparan sulfate proteoglycan 2 (HSPG2) protein, fatty acid binding protein 4 (FABP4) protein, cellular communication network factor 5 (CCN5) protein, RELT TNF receptor (RELT) protein, collagen triple helix repeat containing 1 (CTHRC1) protein, complement factor H (CFH) protein, collagen type VI Alpha 3 chain (COL6A3) protein, angiotensin converting enzyme 2 (ACE2) protein, paraoxonase 3 (PON3) protein.

**Figure 9:**
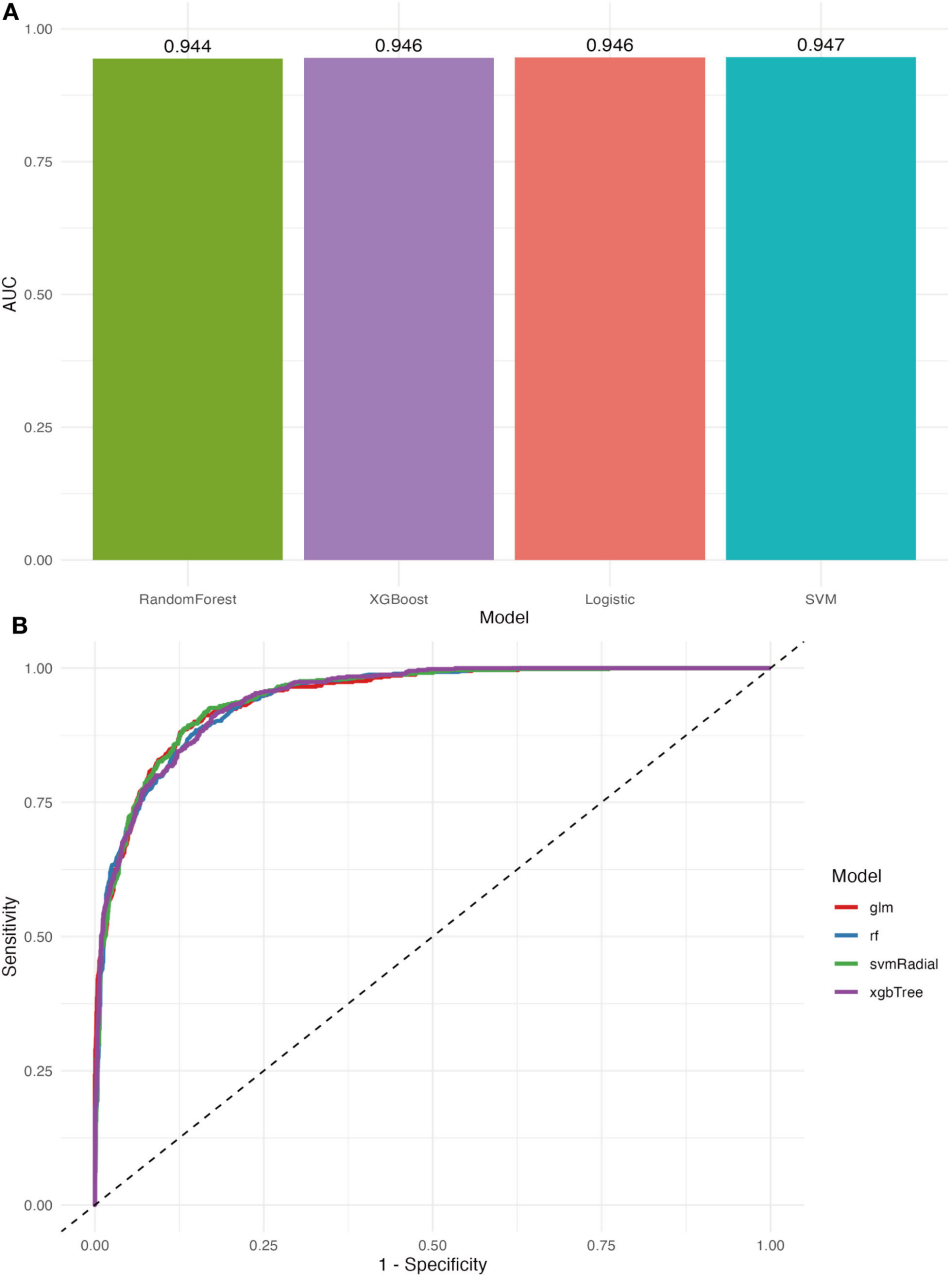
The predictive performance of the 18 proteins using four machine learning algorithms. **(A)** AUC comparison of 18 proteins using 4 methods. **(B)** ROC curve comparison of 18 proteins using 4 methods.

**Figure 10:**
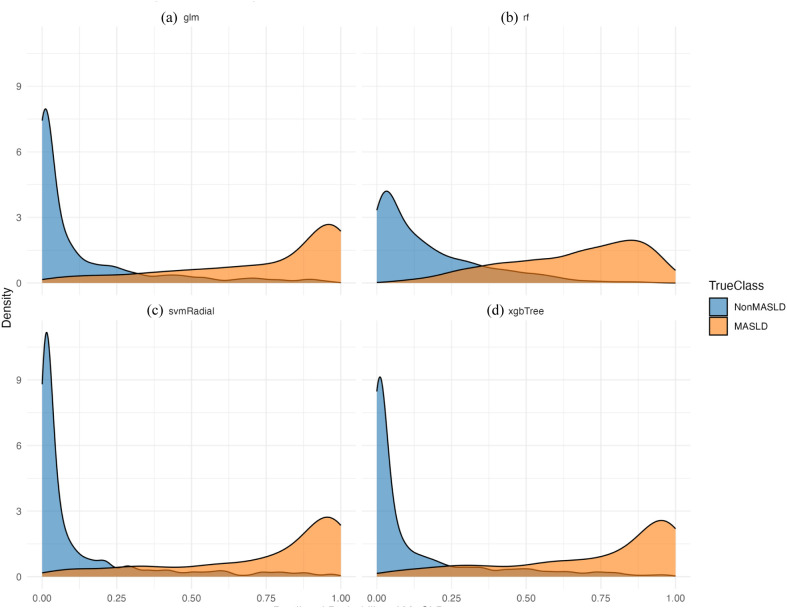
The final predicted probability distribution generated by the 18 proteins.

The diagnostic efficacy of the proteins was verified using the ROC curves and predicted probability distributions in the validation dataset. **Figure 9** displays the AUC values achieved by the 18-protein model using four different machine learning algorithms: RandomForest (AUC = 0.944), XGBoost (AUC = 0.946), Logistic Regression (AUC = 0.946), and SVM (AUC = 0.947). Among the four models, the RandomForest model demonstrated superior discriminatory power, exhibiting minimal distributional overlap between the two groups (**Figure 10**). Furthermore, the predicted probability distributions of all four methods derived from the 18-protein model showed a clearer separation between MASLD and non-MASLD groups, with substantially less overlap compared to the 145-protein model. This indicates superior discriminatory performance of the more parsimonious 18-protein model. Both in terms of statistical performance and practical applicability, the 18-protein model outperformed the full 145-protein model.

To make our finding accessible to clinical practitioners, researchers, patients, and their families, we have created a MASLD risk predictive tool based on these 18-protein model, this tool can be accessed on the following website: https://masldriskpredictor.shinyapps.io/MASLDpredictor/.

## Discussion

In this study, lower circadian relative amplitude (RA) was associated with a higher prevalence of MASLD. Even after adjusting for common sociodemographic and metabolic covariates, a strong association between RA and MASLD prevalence persisted.

Reduced RA—reflecting diminished contrast between activity levels during wake and sleep periods—is a manifestation of circadian rhythm (CR) disruption. This attenuation may result from decreased activity during waking hours (physical inactivity), increased activity during rest (sleep disruption), or both. CR dysregulation elevates the risk of metabolic disturbances, thereby promoting metabolic disorders.

Circadian amplitude has evolved under selective pressures for energy conservation and efficiency, enabling organisms to synchronize their internal clocks with environmental cues, offering significant adaptive benefits (22, 23). Early biological clocks likely served as timekeepers, aligning wakefulness with favorable conditions, driven by cyclic changes in light, temperature, humidity, and other environmental factors linked to Earth’s rotation (24, 25). In mammals, the circadian amplitude is regulated by external cues and endogenous processes, primarily controlled by CLOCK and BMAL1, with the master clock in the suprachiasmatic nucleus (SCN) (22, 26).

However, rapid societal changes, such as artificial lighting, constant temperatures, and sedentary lifestyles, have disrupted circadian rhythms, contributing to pathologies like psychiatric, metabolic, and immune disorders, as well as increased cancer risk (5). This misalignment is strongly associated with metabolic diseases (27).

While complex computational models have been used to quantify circadian disruption, they are often impractical for large-scale datasets due to their complexity and reliance on inaccessible parameters. More practical methods, such as core body temperature monitoring, ECG-derived estimates, sleep timing assessments, and activity level monitoring, require specialized instruments and may not be easily accessible for routine use in large-scale settings (13, 28, 29). In this study, we used RA measures derived from wearable devices, as a key sign of circadian disruption, following previously described protocols. RA is a non-invasive and behaviorally modifiable metric, that has been widely used in previous experiments.

While previous studies have indicated that circadian disruption (e.g., chronic misalignment of sleep–wake cycles) impairs key metabolic regulators such as NAD^+^ and SIRT1 deacetylase activity, suppressing CLOCK/BMAL1 function and leading to impaired insulin secretion and insulin resistance (22, 26, 27, 30, 31), these indicators are primarily suited for cellular and tissue-level studies and are not directly applicable to clinical use. To bridge this gap, we leveraged the UK Biobank’s plasma proteomics data to explore the molecular pathways linking RA and MASLD. Our proteome-wide association study identified 145 proteins associated with both circadian disruption and MASLD. Further correlation and mediation analyses reduced this to 18 key proteins. Functional clustering revealed: 4 proteins (LDLR, LPL, FABP4, LEP) involved in lipid metabolism and transport that may reduce lipid uptake and inhibit lipogenesis; 6 proteins (GDF15, GGT1, ADM, PON3, RELT, CFH) linked to inflammation and immunity that modulate hepatocyte injury; and 5 proteins (COL6A3, CTHRC1, HSPG2, OPTC, CCN5) involved in ECM remodeling and fibrosis. Additional proteins, such as ACE2, play broader roles in metabolic regulation and fibrosis, while others (e.g., NCAN, ADGRG2) remain to be fully understood.

Interestingly, many of these proteins also play roles in the ocular retina. For instance, COL6A3 is upregulated in diabetic retinopathy, while CCN5 inhibits retinal fibrotic changes, and OPTC demonstrates anti-angiogenic activity (32–34). Given that retinal ganglion cells are key photoreceptors in circadian regulation, we hypothesize that these retinal proteins may influence MASLD prevalence by participating in light-mediated circadian signaling (2).

Despite the valuable findings, there are several limitations that should be considered. Participants in the UK Biobank are aged 40–69 years, limiting generalizability to other age groups. In addition, the underrepresentation of non-White ethnic groups may reduce the accuracy and generalizability of ethnicity-specific findings. Although circadian rhythm disruption, as measured by RA, is an effective and convenient indicator, its manifestation can be influenced by multiple factors, including decreased activity during waking hours, increased activity during rest (such as shorter sleep duration or poor sleep patterns). Therefore, future studies should consider using more sleep and activity indices to assess circadian rhythm disruption. While our study has found that RA is independently associated with MASLD, the causal relationship between RA and MASLD remains unclear and requires further investigation. Moreover, the mechanisms by which RA influences MASLD via multiple proteins and the interactions among these factors and their relative contributions, remain unclear and require further validation.

## Conclusion

Our findings support a robust association between circadian disruption and increased MASLD prevalence. We propose activity-derived relative amplitude as a practical biomarker for MASLD susceptibility and identify 18 plasma proteins as potential biomarkers and therapeutic targets. We further develop an accurate, cost-effective predictive model and an accompanying web tool to facilitate early detection and intervention strategies, which may guide lifestyle therapies and monitor treatment response.

## Data Availability

The data used in this study were obtained from the UK Biobank under application number 81434. UK Biobank data are available to bona fide researchers upon application to the UK Biobank [https://www.ukbiobank.ac.uk], subject to approval and compliance with the relevant data access policies. The prediction model developed in this study has been implemented as a publicly accessible web-based tool at [https://masldriskpredictor.shinyapps.io/MASLDpredictor/], which allows users to generate risk predictions based on manually entered variables. The web application does not access, store, or process any individual-level UK Biobank data and operates independently of the UK Biobank database.

## Glossary

MASLD: Metabolic Dysfunction-Associated Steatotic Liver Disease
RA: Relative Amplitude
KEGG: Kyoto Encyclopedia of Genes and Genomes
PPI: protein–protein interaction
SVM: Support Vector Machine
OR: Odds Ratio
CI: Confidence Interval
AUC: Area Under the Curve
ROC: Receiver Operating Characteristic
CR: Circadian Rhythm
SCN: suprachiasmatic nucleus
NAFLD: Nonalcoholic fatty liver disease
UKB: UK Biobank
M10: the most active 10-hour period
L5: the least active 5-hour period
UKB-PPP: UK Biobank Pharma Proteomics Project
FLI: fatty liver index
WC: waist circumference
BMI: Body Mass Index
GGT: gamma-glutamyl transferase
TG: triglyceride
SD: standard deviation
TDI: Townsend Deprivation Index
DEPs: differential expressed proteins
LASSO: Least Absolute Shrinkage and Selection Operator
SBP: Systolic Blood Pressure
DBP: Diastolic Blood Pressure
HbA1c: Hemoglobin A1c
LDL-C: Low-Density Lipoprotein Cholesterol
CRP: C-reactive Protein
HDL-C: High-Density Lipoprotein Cholesterol
T2DM: Type 2 Diabetes Mellitus
NCAN: neurocan
GGT1: gamma-glutamyl transferase 1
ADM: adrenomedullin
LEP: leptin
LDLR: low density lipoprotein receptor
OPTC: opticin
LPL: lipoprotein lipase
GDF15: growth differentiation factor 15
CCN5: cellular communication network factor 5
RELT: RELT TNF receptor
CTHRC1: collagen triple helix repeat containing 1
CFH: complement factor H
COL6A3: collagen type VI Alpha 3 chain
ACE2: angiotensin converting enzyme 2
PON3: paraoxonase 3
